# Co-circulation of Four Human Coronaviruses (HCoVs) in Queensland Children with Acute Respiratory Tract Illnesses in 2004

**DOI:** 10.3390/v4040637

**Published:** 2012-04-23

**Authors:** Ian M. Mackay, Katherine E. Arden, David J. Speicher, Nicholas T. O’Neil, Peter K. McErlean, Ristan M. Greer, Michael D. Nissen, Theo P. Sloots

**Affiliations:** 1 Queensland Paediatric Infectious Diseases Laboratory, Queensland Children’s Medical Research Institute, Sir Albert Sakzewski Virus Research Centre, Children’s Health Service, University of Queensland, Herston 4029, Australia; Email: katherine.arden@uq.edu.au (K.E.A.); d.speicher@griffith.edu.au (D.J.S.); n.oneill@uq.edu.au (N.T.O’N.); p-mcerlean@northwestern.edu (P.K.M.); Michael_Nissen@health.qld.gov.au (M.D.N.); t.sloots@uq.edu.au (T.P.S.); 2 Clinical Medical Virology Centre, School of Chemistry and Molecular Biosciences, University of Queensland, Herston 4029., Australia; 3 Molecular Basis of Disease research Program, Griffith Health Institute, Griffith University, Queensland 4222, Australia; 4 Division Allergy-Immunology, Northwestern University, The Feinberg School of Medicine, Chicago, IL 60611, USA; 5 Queensland Children’s Medical Research Institute, University of Queensland, Herston 4029, Australia; Email: r.greer@uq.edu.au; 6 Division of Microbiology, Pathology Queensland Central, Clinical and Scientific Services, Royal Brisbane Hospitals Campus, Herston 4029, Australia

**Keywords:** respiratory virus, coronavirus, HCoV-HKU1, HCoV-NL63, HCoV-229E, HCoV-OC43, real-time PCR, clinical impact

## Abstract

Acute respiratory illnesses (ARIs) with unconfirmed infectious aetiologies peak at different times of the year. Molecular diagnostic assays reduce the number of unconfirmed ARIs compared to serology- or culture-based techniques. Screening of 888 inpatient and outpatient respiratory specimens spanning late autumn through to early spring, 2004, identified the presence of a human coronavirus (HCoV) on 74 occasions (8.3% of all specimens and 26.3% of all respiratory virus detections). Prevalence peaked in August (late winter in the southern hemisphere) when they were detected in 21.9% of specimens tested. HCoV-HKU1 and HCoV-OC43 comprised 82.4% of all HCoVs detected. Positive specimens were used to develop novel reverse transcriptase real-time PCRs (RT-rtPCRs) for HCoV detection. An objective clinical severity score was assigned to each positive HCoV patient. Severity scores were similar to those from a random selection of young children who were positive for respiratory syncytial virus at a different time but from the same specimen population. During the cooler months of 2004, sensitive and specific RT-rtPCRs identified the concurrent circulation of all four HCoVs, a quarter of which co-occurred with another virus and most of which were from children under the age of two years.

## 1. Introduction

Acute respiratory illnesses (ARIs) are a frequent cause of paediatric morbidity and a common reason for outpatient visits and hospitalisations. Among children, RNA viruses are the most frequent cause of “colds” and “influenza-like” illness (ILIs); usually self-limiting upper respiratory tract illnesses (URTIs) [1]. Virus detections are also often associated with lower respiratory tract illness (LRTI; [2]) although their replication in these tissues is seldom identified. Human rhinoviruses (HRV), respiratory syncytial virus (HRSV), influenzaviruses (IFVs) adenoviruses (HAdV), metapneumovirus (HMPV) and parainfluenza viruses (HPIV) are among the most frequently sought respiratory viruses in the clinical microbiology laboratory [3,4,5,6,7]. Many peak at distinct times of the year. However, even when bacterial pathogens are added to this viral panel, 40–70% of suspected infections remain without laboratory confirmation [8,9,10]. The inclusion of more viruses into the diagnostic algorithm is recommended to support and confirm a clinically diagnosed infectious aetiology [11]. Nonetheless, extended testing panels are most often used by research projects and the human coronaviruses (HCoVs) are one group of pathogens often overlooked by routine testing. Two of the enveloped positive sense RNA HCoVs, HCoV-229E and HCoV-OC43, have been known for more than 40 years [12,13]. Infections by these two viruses can be difficult to distinguish from ILI in a population vaccinated for IFV [14]. Both HCoVs have also been identified in non-respiratory specimens [15]. Recently three new HCoVs, all detected in patients with ARIs were described; the severe acute respiratory syndrome coronavirus (SARS-CoV) in 2003, HCoV-NL63 in 2004 and HCoV-HKU1 in 2005 [16,17,18].

Some studies have noted that genus *Alphacoronavirus* species HCoV-NL63 and HCoV-229E and the genus *Betacoronavirus* species HCoV-HKU1 and subspecies HCoV-OC43 may circulate annually, but the prevalence varies significantly [19]. Only HCoV-HKU1 has a seroprevalence below 90% in adults; the reason for this apparently reduced exposure may be related to a cross-protective antibody effect afforded by prior infection with HCoV-OC43 [20,21].

RT-PCR methods more frequently detect HCoVs than *in vitro* culture because they are difficult to grow without a source of primary tissue [22,23,24]. Studies that definitively link the presence of viral RNA to human disease, akin to those conducted using infection of human volunteers are lacking. This has hampered the assignment of specific disease associations for these and other newly identified respiratory viruses. Screening for all HCoVs in a multi-year population sampling is infrequent so studies that directly compare the impact of HCoV infections are rare [19,25,26,27,28,29,30,31]. Despite a sizable historical role in the common cold [32] the HCoVs are also found in patients with more severe cases of ARI [26,33,34,35] including LRTI and pneumonia in adults and the elderly [9,36,37]. HCoVs are also found in cases of bronchial hyper-responsiveness in susceptible individuals [38,39] and nosocomial respiratory viral infections [40,41,42].

We have previously identified instances of HCoV-HKU1 from samples collected during the winter of 2004 [43]. Here we expanded upon that investigation, using newly designed reverse transcriptase real-time PCR (RT-rtPCR) assays to screen for all four non-SARS-CoVs in a hospital-based, predominantly paediatric population with ARI. The clinical status of patients with single and multiple HCoV detection was also compared.

## 2. Results and Discussion

### 2.1. Detection of HCoVs and RT-rtPCRs

We expanded our previous study of 324 specimens using PCR assays to screen for HCoVs. We previously identified that a pan-HCoV RT-PCR often failed to detect HCoV-HKU1 and HCoV-OC43 although it was useful for HCoV-229E and HCoV-NL63 detection [43,44]. We therefore included a specific HCoV-HKU1 assay [45] and developed an RT-rtPCR for HCoV-OC43 detection (this study). When enough specimen extract remained, positives were confirmed using nucleotide sequencing (Figure 4).

Newly developed HCoV-HKU1, HCoV-NL63 and HCoV-229E RT-rtPCRs confirmed previous and new (Table 1; this study) conventional RT-PCR HCoV positives [43,46]. Previous sequencing of the 1b region had confirmed HCoV identity where specimen remained [43,47]. The RT-rtPCRs did not produce any positive results (defined in the Experimental section) when amplifying clinical samples positive for HRSV (n = 103), HAdVs (n = 26), HRVs (n = 17) IFVs (n = 5) or HPIV-positive (n = 28) specimens. The HCoV RT-rtPCRs have since been used in several other studies (including many more positives for each of these respiratory viruses listed) and no cross-reactions have been noted. The analytical sensitivity of each assay was determined to be ≤10^1^ copies ivtRNA/20 μL reaction.

Overall 74 instances of a coronavirus (8.3% of tested specimens) were detected. The majority of which were HCoV-HKU1 (n = 34; 46.6% of all HCoV), followed by HCoV-OC43 (n = 27; 37.0%), HCoV-NL63 (n = 9;12.3%) and HCoV-229E (n = 4; 5.5%). 

### 2.2. Epidemiology and Clinical Features of HCoV-positive Individuals During Winter 2004

**Figure 1:**
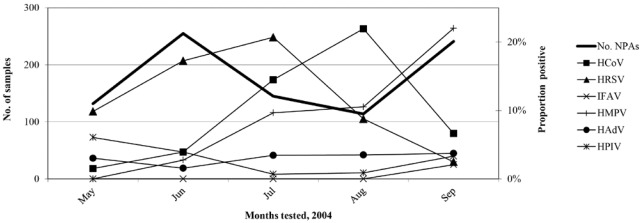
Specimens tested and the proportion of respiratory viruses positive during the study period.

HCoV detection peaked in late winter (August in the southern hemisphere) when sample numbers were lowest (Figure 1). Most (69.9%) of the HCoV detections were from patients two years old or less with children (age ≤ 14 years) comprising 80.8% of the positive patients. HCoV types were equally distributed between males and females except for HCoV-NL63 which was only detected in males (p = 0.012; 95% confidence interval). HCoV-OC43 was detected in all age groups, was the only HCoV detected in neonates (n = 2) and was detected in adults (6.3% of all viruses detected in patients over 14 years old) more than any other respiratory virus (≤3.1%). 

**Figure 2:**
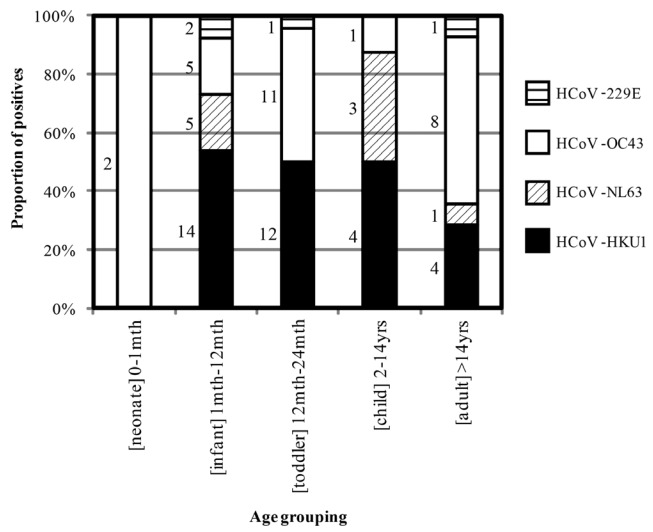
Comparison of the proportion of each HCoV detected in each age group. The number of HCoV detections is shown to the left of each bar.

The average age of positive patients was highest for HCoV-OC43 among the four HCoVs (Figure 2; Table 2). Each HCoV was involved in more co-detections than the average level of co-detections for the study population (10.5%; Table 2) and more frequently (HCoV-229E, 25.0%; HCoV-OC43, 11.1%; HCoV-HKU1, 14.7%) than most other respiratory viruses sought (HRSV, 4.9%; HAdV, 15.4%; HPIV3, 28.6%; HMPV, 8.1%).

Clinical reviews were obtained on 62 HCoV positives, representing 61 patients (83.6% of HCoV detections; Table 3). In one patient both HCoV-HKU1 and HCoV-OC43 were co-detected (sequencing confirmed that both viruses were present; data not shown). This male toddler (13 months old), also positive for HMPV, had been admitted three months prior with bronchiolitis. For the current presentation, following seven days of symptoms including cough, otitis media, a measurable fever and rhinorrhoea, salbutamol and antibiotics were administered. 

**Figure 3:**
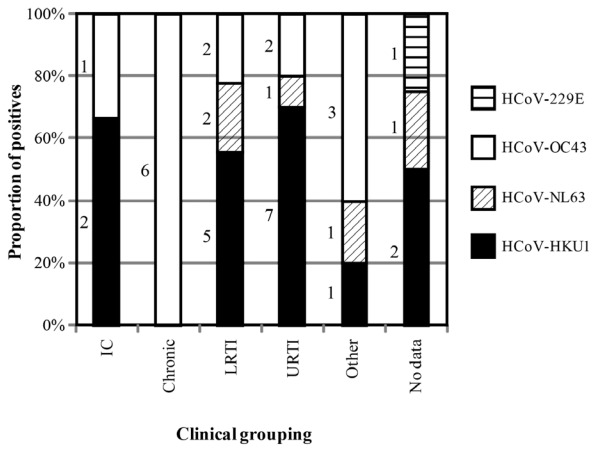
Clinical groupings among the single detection of each HCoV. Based on the criteria of Gaunt *et al.* [19]. The number of HCoV detections is shown to the left of each bar. IC-immunocompromised; LRTI-lower respiratory tract illness; URTI-upper respiratory tract illness.

The average clinical severity score among patients positive for any HCoV was 2.1 with 47 cases (77.0% of HCoV-positive reviews) admitted to hospital (26 were only positive for an HCoV). Of these admissions, 20 (32.8%) remained for ≥96 hours, with 14 of these sole HCoV detections. There was no difference in severity score between the four HCoV groups, either between samples with a single detection and co-detections considered together, p = 0.21, or between those with single detections, p = 0.29, or those with co-detections, p = 0.09 (Kruskal-Wallis test). The highest average severity score (4.5) was observed from HCoV-NL63-positive patients who were also positive for another virus. Most HCoV cases met Gaunt *et al.*’s criteria [19] for URTI (n = 17; 27.4%) or LRTI (n = 19; 30.6%; Table 2). Data could not be obtained for four (6.5%) HCoV-positive cases. Antibiotics were given to 22 cases (36.1%). HCoV-HKU1 detections, whether single or co-detections, were most often made from patients with URTI or LRTI (70.9%; Figure 3). Most single HCoV-OC43 detections were in patients with chronic disease (38.1% of detections). Most HCoV-229E detections were accompanied by another virus and no HCoV-NL63 detections were made in chronically diseased or immunocompromised patients.

The most commonly noted additional clinical features among patients only positive for a HCoV (n = 37; 59.7% of HCoV cases) were cough (n = 6; 9.7%), fever (n = 5; 8.1%) and vomiting (n = 4; 6.5%; Table 3). Among HCoV co-detections the most common features were otitis media (n = 5; 8.1% of HCoV cases), cough (n = 4; 6.5%), fever (n = 3; 4.8%) and vomiting (n = 3; 4.8% of HCoV-positives). 

In the comparison population of HRSV-positive children whose charts were reviewed the average severity score was also 2.1. The obvious differences between HRSV and HCoV hospitalisations were that slightly fewer HRSV cases were admitted than in the HCoV group (70.0% *vs. *77.0%), hospitalisation times were shorter (20.0% *vs. *32.8% inpatients at 96 hours) and fewer patients required mechanical ventilation (5.0% *vs. *11.5%). However, more HRSV-positive children had a measurable fever (n = 13; 65% compared to 10.3% of HCoV-positive patients).

**Table 1:** Oligonucleotide primers and probes used for HCoV screening.

Oligonucleotide Name (gene target)	Oligonucleotide Sequence
229E 01.4 (N)	ACAACGTGGTCGTCAGGGT
229E 02.6 (N)	GCAACCCAGACGACACCT
229E_MGB	FAM-CATCTTTATGGGGTCCTG -MGBNFQ
229E 01.3 T7	AAAA**TAATACGACTCACTATAGGG**GAACCACAACGTGGTCGTCAGGGT
229E 02.5 T7	GGTTCTGAATTCTTGCGCCTAA
HKU1 01.2 (1b)	GTTGGGACGATATGTTACGTCATCTT
HKU1 02.2 (1b)	TGCTAGTACCACCAGGCTTAACATA
HKU1_MGB	FAM-CAACCGCCACACATAA-MGBNFQ
HKU1 01.2 T7	AAAA**TAATACGACTCACTATAGGG**GTTGGGACGATATGTTACGTCATCTT
HKU1 02.2 T7	TGCTAGTACCACCAGGCTTAACATA
OC43 01.3 (N)	GAAGGTCTGCTCCTAATTCCAGAT
OC43 02.4 (N)	TTTGGCAGTATGCTTAGTTACTT
OC43_TM	ROX-TGCCAAGTTTTGCCAGAACAAGACTAGC-BHQ2
OC43 01.2 T7	AAAA**TAATACGACTCACTATAGGG**CGATGAGGCTATTCCGACTAGGT
OC43 02.4 T7	ACCAGATGCCGACATAAGGTTCATTCT
NL63_N_01.13 (N)	GAGTTCGAGGATCGCTCTAATA
NL63_N_02.8 (N)	TGAATCCCCCATATTGTGATTAAA
NL63_TM5	CY5-AAAATGTTATTCAGTGCTTTGGTCCTCGTGA-BHQ1
NL63 01.6 T7	AAAA**TAATACGACTCACTATAGGG**AGTCTTGGTAATCGCAAACGTAATC
NL63 02.6 T7	TATCAAAGAATAACGCAGCCTGATTA

01-sense primer; 02-antisense primer; T7–primers used to make ivtRNA controls showing the promoter region (bold, underlined) and 5’ spacer sequence (underlined, not bold).

**Table 2:** Patient population and HCoV findings.

	229E	NL63	OC43	HKU1	Total population (n = 888)
Male (%)	2 (50.0)	9^1^ (100)	12 (44.4)	19 (55.9)	514 (57.9)
Detections	4	9	27^2^	34^2^	290^3^ (32.7)
Co-detections (%)	3 (75.0)	2 (22.2)	6 (18.5)	11 (23.5)	29^4^ (10.5)
Mean age, years	4.9	3.9	18.6	4.9	7.9
Peak month	July	August	July	August	June
Average severity score (single/co-detection)	ND/2.5	2.0/4.5	2.9/2.0	1.6/1.5	-

^1^p=0.012 with 95% confidence level^ 2^A single co-detection between HCoV-OC43 and HCoV-HKU1 are included in each tally; ^3^Total of all virus detections including HCoVs; ^4^-only HCoV-positive samples were screened for HRVs; ND-no data available

**Table 3:** Clinical features of patients positive for an HCoV.

HCoV detected	Average Score ^1^	Detections	Fever	Vomit	Cough	Diarrhoea	Rash	AOM ^2^	IC ^3^ (n = 4)	chronic (n = 9)	LRTI (n = 18)	URTI (n = 17)	other (n = 9)	no data (n = 4)
HKU1 single	1.63	17	3	3	3	0	2	2	2	0	5	7	1	2
HKU1 dual	1.54	14	1	3	2	1	1	3	1	1	4	6	2	0
NL63 single	2.00	5	0	0	2	0	0	0	0	0	2	1	1	1
NL63 dual	4.50	2	0	0	0	0	0	0	0	0	2	0	0	0
OC43 single	2.85	14	2	1	1	0	0	0	1	6	2	2	3	0
OC43 dual	2.00	7	2	0	2	0	0	2	0	2	2	1	2	0
229E single	ND	1	0	0	0	0	0	0	0	0	0	0	0	1
229E dual	2.50	2	0	0	0	0	0	0	0	0	2	0	0	0
		62	8	7	10	1	3	7	4	9	19	17	9	4
	SoDe HCoV		5	4	6	0	2	2	3	6	9	10	5	4
			8.1%	6.5%	9.7%	0.0%	3.2%	3.2%	4.8%	9.7%	14.5%	16.1%	8.1%	6.5%
	CoDe HCoV		3	3	4	1	1	5	1	3	10	7	4	0
			4.8%	4.8%	6.5%	1.6%	1.6%	8.1%	1.6%	4.8%	16.1%	11.3%	6.5%	0.0%

^1^ The severity score is a 5-point index which takes oxygen requirement to be the best single quantifier of respiratory illness. Additional component scores are derived from the need for admission, intravenous fluids and the time until discharge. This peer-reviewed severity index has been validated during previous studies [48,49,50]. ; ^2^ acute otitis media; ^3^ immune compromise

**Figure 4:**
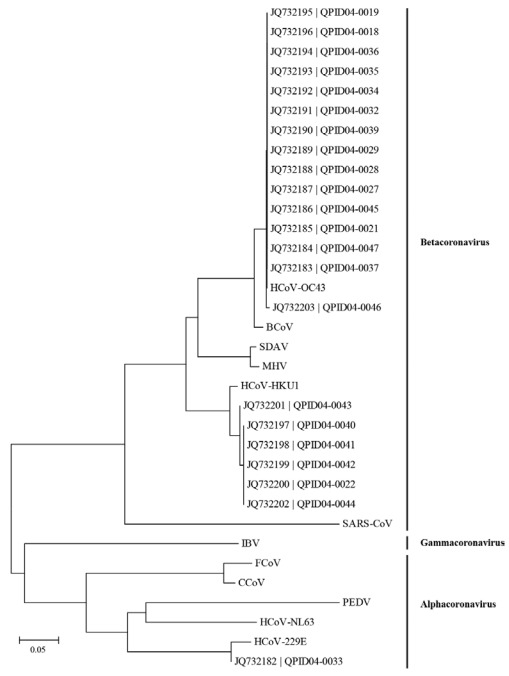
**Confirmation of HCoV Identities**. Phylogenetic analysis of some Queensland HCoVs detected during the winter of 2004 from cases of ARI, presented on a topology tree prepared in MEGA 5 and compared to other coronavirus sequences (BCoV, DQ811784-bovine coronavirus, assigned ot the same species as HCoV-OC43; PEDV, NC_003436-porcine epidemic diarrhoea virus; MHV, NC_001846-murine coronavirus; CCoV, AF124986-canine coronavirus; FCoV, AF124987-feline coronavirus; IBV, NC_001451-avian infectious bronchitis virus; SDAV, AF124990-; HCoV-NL63, AY567487; HCoV-229E, AF304460; HCoV-OC43, NC_005147; HCoV-HKU1, NC_006577; SARS-CoV, AY274119). The nucleotide alignment of 225nt of the polymerase gene was prepared using Geneious Pro v5.5. The tree was drawn to scale and evolutionary distances were calculated using the Maximum Composite Likelihood method. HCoV groupings are indicated.

## 3. Experimental Section

### 3.1. Study Population

The study comprised 888 respiratory specimens from individuals who had presented to Queensland hospitals with signs and symptoms of ARI during May to September 2004. This included 324 previously described specimens [43]. Specimens were predominantly nasopharyngeal aspirates (NPA; 97.9%) collected either from outpatients or admitted patients. Specimens were selected by season, without prior knowledge of patient details or viral diagnostic status. The subjects ranged in age from three days to 92.4 years (mean = 7.9 years, median = 1.1 years, mode = 0.2 years), with infants (one to twelve months old) comprising 41.1% of the study population. 

Prior to this retrospective study, nucleic acids had been extracted from specimens, tested for common respiratory viral pathogens and stored at -70°C. The clinical microbiology laboratory assays included culture-amplified direct fluorescent assay and subsequent PCRs to detect HRSV, HAdV, HPIV-1, 2 and 3 and influenza viruses A & B (IFAV, IFBV) [8]. An additional PCR assay was used to detect HMPV [51]. No virus was detected in 574 specimens (64.6%). Additional RT-rtPCR testing for human rhinoviruses (HRVs) was based on a modified, previously described method [52,53], only applied to the HCoV-positive samples.

### 3.2. HCoV RT-PCR Testing

The RT-PCRs used to initially screen specimens included a mix of conventional (for HCoV-229E, HCoV-NL63 and HCoV-HKU1; described previously [43,54]) and RT-rtPCRs (HCoV-OC43; described in this study). The positives patients from these assays were subject to retrospective medical chart review. The positive specimens, when extract remained, were used for the validation of newly designed RT-rtPCRs for the detection of HCoV-229E, HCoV-NL63 and HCoV-HKU1.

Single-target RT-rtPCR assays were developed to maximise sensitivity, using in-house oligonucleotides specific to HCoV-229E, HCoV-HKU1, HCoV-OC43 or HCoV-NL63 (Table 1). Reaction mixes were combined with 1 μL of purified RNA and subjected to RT (OneStep RT-PCR kit, QIAGEN, Australia) for 30 min at 50 °C followed by a 15min incubation at 95°C. PCR was performed for 55 cycles of 94 °C for 5 sec, 60 °C for 60 sec (Rotor-Gene 3000, 6000 or Q, QIAGEN, Australia). Synthetic T7-tagged HCoV-specific *in vitro* transcribed RNAs (ivtRNAs) were created by adapting a previously described approach [55]. We used oligonucleotides which bound at (HCoV-HKU1) or outside (all other HCoVs) the diagnostic RT-rtPCR target area (T7 oligonucleotides; Table 1). RNA was subjected to two treatments with DNase (Turbo, Life Technologies) followed by column purification (High Pure Viral Nucleic Acid Kit, Roche Diagnostics). These stock RNAs were used to determine the analytical sensitivity of each assay after testing a 10-fold dilution series. The last dilution to yield a positive result (defined below) was taken as the limit of analytical sensitivity. The ivtRNA copy numbers were calculated using the optical density to approximate the mass of RNA in the stock solution at 260 nm, determining the number of moles by establishing the molecular weight of each HCoV-specific ivtRNA and then multiplying by 6.02 × 10^23^ (Avogadro’s number). A positive diagnostic result was defined by the presence of a sigmoidal curve that crossed an arbitrary threshold of 0.05, before 45 cycles, during an experiment in which duplicate non-template controls did not cross the threshold.

### 3.3. Nucleotide Sequencing and Phylogenetic Analysis

Nucleotide sequencing reactions were performed using 2 *μ*L of amplicon with the ABI PRISM™ BigDye cycle sequencing kit (Perkin Elmer Applied Biosystems Division, USA). Sequences were determined using an Applied Biosystems 3130xl capillary electrophoresis genetic analyser.

Nucleotide sequences were aligned using Geneious Pro v5 [56] and presented in a neighbour-joining tree prepared in MEGA 5 [57]. Nucleotide sequences deposited into GenBank as a result of this study include JQ732182-JQ732203.

### 3.4. Clinical Data Collection

Medical records of patients with specimens positive for HCoV by conventional RT-PCRs [43,54] were reviewed to determine the severity of the ARI using a validated, objective scoring system [49]. The severity score is based on the patient’s need for hospitalisation, supplemental fluids and degree of respiratory support. If other clinical information such as cough, wheeze, fever and pre-existing conditions was documented, this was also recorded. A secondary dataset of clinical information (n = 20) was also collected from age-matched patients (less than three years of age) positive for HRSV who had also presented with ARI between July and December 2009.

## 4. Discussion

We retrospectively investigated an Australian paediatric, hospital-based winter population for the presence of HCoVs and developed four novel RT-rtPCRs. Studies identifying the co-circulation of all four non-SARS-CoVs are uncommon [19,25,26,27,28,29]. A paucity of such data hinders direct comparison of the epidemiology and clinical features of individuals infected by the different HCoVs. Some assays designed to detect all four HCoVs are too insensitive for practical use in a clinical microbiology laboratory [44,58]. Others, using similar measures of assay sensitivity to those employed here, have proven sensitive and specific when used to screen focused or hospitalized disease populations [30,59,60]. The appearance of commercial assay alternatives is progressing but large scale or routine use is rare [61]. The incidence of 8.3% in our samples is higher than reported by a three-year study (<1% prevalence; [19]) and among those hospitalized with pneumonia or ARI and/or fever [30,59,60] reflecting our more focused winter study period and mixed inpatient and outpatient hospital population; the known period of peak HCoV prevalence. Similar to others, we observed a high number of co-detections (18.5–75.0%) among the HCoVs compared to the overall study population (10.5%) [19,62]. Others have commented that HCoVs impart a limited impact due to their low prevalence, relatively higher proportion of involvement in co-detections and low secondary attack rate in households [62]. Our retrospective study also observed that clinical features and the average severity scores from patients with HCoV were very close to those from a similarly aged set of children who were positive for HRSV. 

HCoV-OC43 was detected in patients with a range of clinical conditions but predominated among those with chronic conditions and manifested as both URTI and LRTI. HCoV-HKU1 dominated detections overall and specifically in those with URTI, LRTI and in those with vomiting, cough, fever and otitis media. Our findings are similar to those from Hong Kong where HCoV-HKU1, previously identified in patients with pneumonia, was associated with fever and febrile convulsion [25]. However, HCoV-HKU1 whether as a single or co-detection had the lowest average severity score, a metric that was focussed on respiratory symptoms. Dijkman *et al.* proposed that HCoV-OC43 seroconversions dominated those of HCoV-HKU1 (and that HCoV-NL63 dominated HCoV-229E) because of cross-protective neutralizing antibodies elicited by the first infection with HCoV-OC43 or HCoV-NL63 [20]. Our study found that most HCoV-HKU1 detections (peaking in August) were made after the HCoV-OC43 detections (peaking in July). Although the numbers in our winter study were smaller than those of Dijkman *et al.*’s and the differences in peak detection were not great, our testing of all inpatient and outpatient samples from Queensland hospital presentations between May and September 2004 may have better resolved and reflected HCoV activity than did the outcome of a five-year average of testing results from paediatric inpatients below the age of two years [20]. Extending serosurveys to other populations may resolve this discrepancy. Neither HCoV-NL63 nor HCoV-229E occurred in patients with rash, immunocompromise, diarrhoea or vomiting or in those with chronic disease. HCoV-NL63 was most common among those with cough (including croup and prolonged cough) and LRTI which is in keeping with its association with croup [5]. Vomiting and diarrhoea occurred most often in HCoV-HKU1 cases supporting other recent findings for this HCoV [63]. 

Several study limitations should be highlighted. This population does not represent clinically well subjects (*i.e.*, those who do not feel ill enough to warrant a visit to their hospital or outpatients clinic) so no conclusions about the impact of these viruses in the community can be drawn beyond the extrapolation that because our patient population is sampled from the community, it is reasonable to assume that these viruses have been circulating concurrently. It is additionally worth noting that all respiratory viruses have so far been found to some extent in clinically well populations. This finding does not bestow unimportance or reduced pathogenicity upon the viruses, just as a statistically significant higher proportion in the number of detections in an ill population versus a control group cannot guarantee causality upon the virus. Because human immunity plays a significant regulatory role in every virus challenge a control population is only the first of many steps to identify a disease association. Another useful step is a birth cohort followed for several years and sampled at regular short intervals regardless of disease state. We have such a cohort underway. Because this is a cross-sectional study, we could not define what was happening to the HCoVs during the “off-season”. We did not test all specimens for bocaviruses or HRVs so the overall number of co-detections may be under-reported.

We did not expect the predominance of males among the HCoV-NL63 cases, a pattern of gender distribution which differs from the other HCoVs. It is possible this is a type 1 error associated with multiple tests. However, as there were only four tests and a small p-value, it is possible this indicates a true virus effect, which requires further investigation for confirmation. HCoV-HKU1 was the most frequently detected HCoV and involved in the broadest array of clinical outcomes. HCoV-OC43 should be considered in the diagnosis of acute adult respiratory disease while HCoV-NL63 may play a role in more severe disease involving multiple viruses. Because HCoV-229E occurs infrequently and mostly in co-detections, its role in ARIs beyond the common cold remains hard to define. Despite reports of a role for HCoV-229E in ARI in immunocompromised patients [19], we did not have many cases of immunocompromise in our population and so we may have missed this role in our study. 

## 5. Conclusions

We have presented the design and application of four sensitive, specific and novel RT-rtPCRs that allow us to differentiate between peak HCoV prevalence and that of other circulating respiratory viruses. We observed HCoV-specific differences in co-detection frequencies, monthly peak prevalence and the sex of the positive patients. Even though many differences were noted in this cross-sectional study, future respiratory virus studies are needed to confirm and better define these differences through frequent analysis of cohortees and/or studies conducted over greater periods of time. Such prospective cohort studies will improve our understanding of the biology of these viruses, and ultimately improve patient care by better defining discordances between testing methods, study timing and population characteristics.
